# The prognostic analysis of further axillary dissection in breast cancer with 1-2 positive sentinel lymph nodes undergoing mastectomy

**DOI:** 10.3389/fonc.2024.1406981

**Published:** 2024-08-05

**Authors:** Xueyi Zhao, Liu Yang, Congbo Cao, Zhenchuan Song

**Affiliations:** Department of Breast Center, The Fourth Hospital of Hebei Medical University, Shijiazhuang, China

**Keywords:** breast neoplasm, sentinel lymph nodes, lymph node dissection, propensity score, prognosis

## Abstract

**Background:**

The ACOSOG Z0011 study has shown that axillary lymph node dissection (ALND) is an option to be considered in patients who had 1-2 metastatic sentinel lymph nodes (SLNs) who proceed with breast-conserving along with postoperative radiotherapy. However, there remains controversy regarding the applicability of this approach in patients who had a mastectomy. The aim of our study is to determine the prognostic differences and risk factors associated with the decision to opt for ALND in breast cancer patients who had 1-2 metastatic SLNs who receive a mastectomy.

**Methods:**

The study conducted a retrospective analysis of patients diagnosed with cT1-2N0 breast cancer and treated at The Fourth Hospital of Hebei Medical University between January 2016 and December 2021, and patients were divided into two cohorts according to whether ALND was performed after sentinel lymph node biopsy (SLNB): SLNB cohort and SLNB + ALND cohort. Outcomes included the locoregional recurrence rate (LRR), disease-free survival (DFS), and overall survival (OS). Propensity score matching (PSM) was conducted to ensure the balance of variables between the two cohorts. Cox proportional hazard models were employed to ascertain the univariate and multivariate relative risks associated with survival.

**Results:**

There were 812 cases enrolled. After the PSM, 234 receiving ALND and 234 not receiving ALND were matched. A median follow-up period of 56.72 ± 20.29 months was observed. During that time, no significant difference was identified in the DFS and OS in the SLNB + ALND cohort and the SLNB cohort (P = 0.208 and P = 0.102), except for those under 40 years old, SLNB + ALND group showed a reduction in LRR compared to SLNB group (11.1% vs. 2.12%, P = 0.044). Multivariate Cox analysis showed that younger (≤ 40 years), progesterone receptor (PR)-negative, and SLNB alone were independent risk factors for LRR; perineural invasion was a risk factor, while endocrinotherapy was a beneficial prognostic indicator for DFS and OS among patients with positive hormone receptor.

**Conclusion:**

ALND does not impact DFS and OS in patients with 1-2 metastatic SLNs who have completed a mastectomy. Being younger (≤ 40 years), having a negative PR, and undergoing SLNB alone were independent risk factors for LRR. Given this finding, we recommend avoiding axillary treatment such as ALND or radiotherapy in patients without risk factors.

## 1 Introduction

It is widely acknowledged that breast cancer is the most highly occurring malignant disease among females, posing significant threats to their health (1, 2). Over the past few years, sentinel lymph node biopsy (SLNB) has become as a prevalent technique for diagnosing the status in the axillary region, aiming to reduce trauma and enhance treatment efficacy (3). SLNB may be a substitute for axillary lymph node dissection (ALND) in patients who have a negative sentinel lymph node (SLN), as evidenced by the NSABP-B32 trial (4–6). The current research agenda in the field of breast cancer surgery includes the question of how to manage the axillary region in cases where the SLN is positive (7). The ACOSOG Z0011 trial has shown that patients who proceed breast-conserving in conjunction with postoperative radiotherapy and present with one or two metastatic SLNs are not likely to benefit from additional axillary surgical intervention (8, 9). The AMAROS and IBCSG23-01 studies have furnished corroborating evidence for the strategic intervention of the axilla in patients exhibiting SLN micrometastases (10). Nevertheless, the conduct of axillary surgery in mastectomy patients with 1-2 metastatic SLNs remains a topic of contention (11).

As a component of breast cancer surgical therapy, ALND diminishes the risk of axillary recurrence and provides valuable insights for guiding subsequent treatment decisions (12, 13). The rates of axillary recurrence and survival outcomes were similar between patients who had ALND and those who had no axillary surgery, as reported by the IBCSG 10-93 trial (14). The findings of the study by Gao et al. indicate the survival advantage associated with ALND in individuals who have received a mastectomy and one or two metastatic SLNs is not statistically significant (15–17). Chen et al. also reported similar findings (18, 19). What methods do surgeons employ to ascertain whether a patient with one or two metastatic SLNs would derive benefit from an ALND?

In addition to survival benefits, the locoregional recurrence rate (LRR) of metastases is an important consideration for surgeons when deciding on the operation. The study conducted by Giuliano et al. indicated that SLN dissection alone (but not ALND) is an effective approach for achieving regional control in those with clinically early-stage breast cancer and receiving breast-conserving surgery followed by adjuvant systemic therapy (20). But in patients who undergo mastectomy, can SLNB achieve the same effect?

Our study aims to explore the effect of ALND on prognosis based on molecular subtypes. Meanwhile, by analyzing the differences in LRR, DFS, and OS in different age groups, we investigated the necessity of ALND.

## 2 Materials and methods

### 2.1 Study population

**Figure 1:**
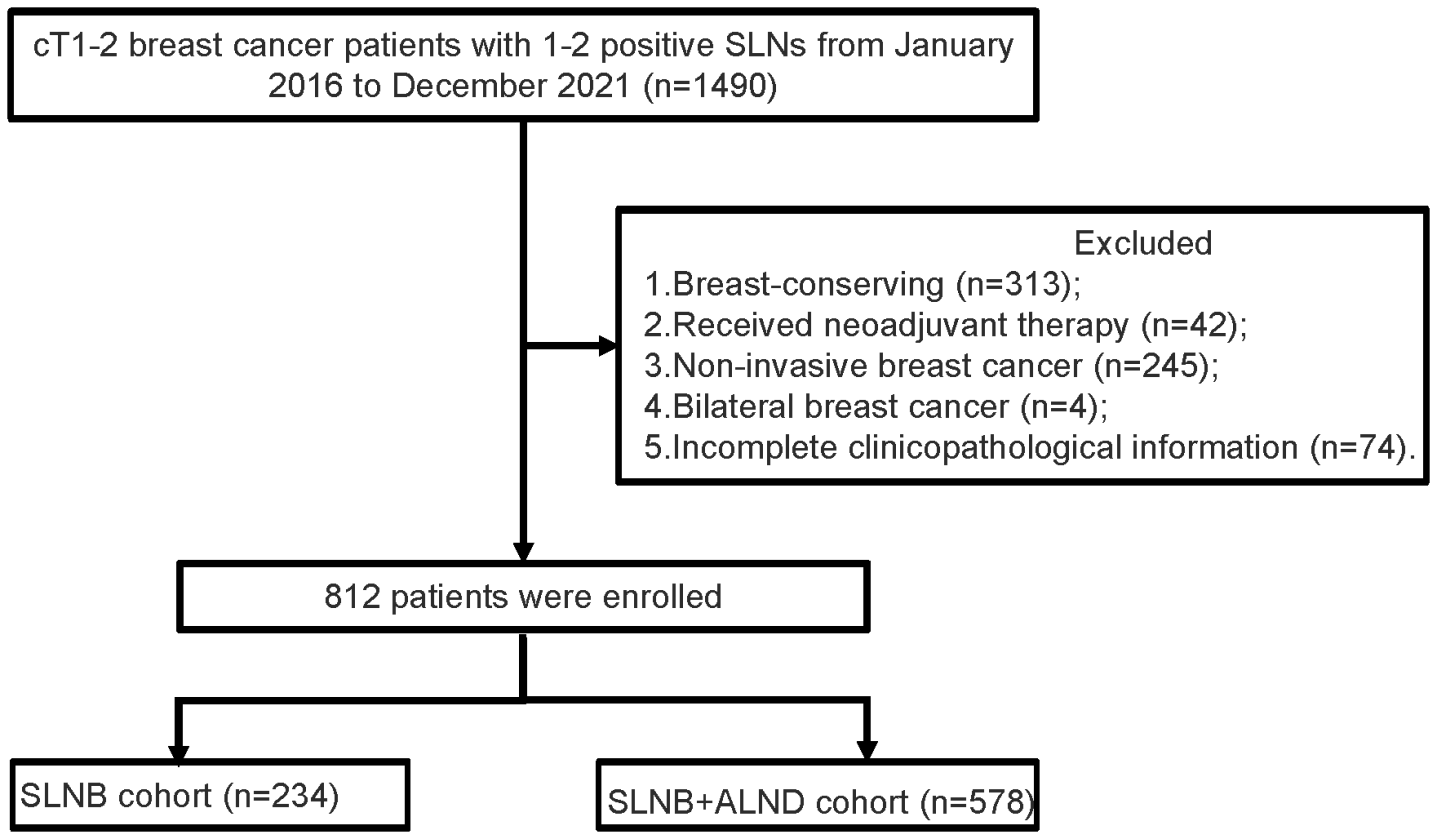
Flowchart of patient selection. SLNs, sentinel lymph nodes; SLNB, sentinel lymph node biopsy; ALND, axillary lymph node dissection.

A retrospective analysis was conducted on the pathologically confirmed breast cancer patients who underwent surgery at The Fourth Hospital of Hebei Medical University from January 2016 to December 2021 (**Figure 1**). Inclusion criteria (1): The patient must be female (2). A diagnosis of breast cancer is defined as occurring at an age of at least 18 years (3). Mastectomy was performed (4). There should be 1-2 macrometastases in the sentinel lymph node (SLN) (5). The paraffin pathology diagnosis should be invasive ductal carcinoma (IDC) or invasive lobular carcinoma (ILC). Exclusion criteria (1): Incomplete clinicopathological information is a reason for exclusion. (2) Having undergone breast-conserving surgery is not included. (3) Patients who have received neoadjuvant chemotherapy or radiotherapy are excluded. (4) Bilateral breast cancer is also among the exclusion criteria. (5) Metastatic breast cancer is an exclusion factor. (6) The existence of other malignant tumors is not allowed.

### 2.2 Data collection

The data was gathered from the medical records of eligible patients, including information of age, surgery of axillary, information on pathological, treatment, and follow-up or death.

### 2.3 Diagnostic criteria and outcome definition

It is worth noting that 20% is used as the cutoff for PR expression levels and serves as the threshold between Luminal A and Luminal B subtypes. Additionally, 14% is used as the threshold for Ki-67 expression levels. Immunohistochemistry (IHC) 3 + or amplified in fluorescence *in situ* hybridization (FISH) was defined as HER2 positive (21).

The primary outcome was the locoregional recurrence rate (LRR), and it was defined as the recurrence occurring in the chest wall, same breast, or regional lymph nodes. The secondary outcomes encompassed disease-free survival (DFS) and overall survival (OS). DFS was characterized as the period starting from the surgery until the date of disease recurrence, death, or the last follow-up. OS was described as the time interval from the surgery to the date of all-cause death or the last follow-up.

### 2.4 Statistical analysis

Continuous variables were expressed as the mean along with the standard deviation. The difference between the groups was examined through the Student t-test. Categorical variables were presented as proportions and tested by Pearson’s chi-square test or Fisher’s exact test.

To balance the significant differences between patients who received ALND and those who did not, propensity score matching (PSM) was performed at a 1:1 ratio using the nearest-neighbor method with a caliper of 0.02. The Kaplan-Meier was used to plot survival curves, and the log-rank test was used to compare survival differences between groups. Univariate and multivariate cox proportional hazard models were used to identify risk factors that were associated with LRR, DFS, and OS.

SPSS 22.0 and R software (version 4.2.2) were used for statistical analysis, along with MSTATA software (www.mstata.com). All tests were two-tailed, and P < 0.05 was considered statistically significant.

## 3 Results

### 3.1 Baseline characteristics

A total of 1490 cases of cT1-2 with 1-2 metastatic SLN and 678 did not meet the inclusion criteria. It were excluded (313 for breast-conserving, 42 for received neoadjuvant therapy, 245 for non-invasive breast cancer, 4 for bilateral breast cancer, and 74 for incomplete clinicopathological information). Finally, 812 patients were finally enrolled.

The mean age of the whole population was 51.83 ± 11.16 years (range 27 to 85). Among them, 310 (38.18%) were Luminal A subtype, 271 (33.37%) were Luminal B, 176 (21.68%) were HER2 positive, and 55 (6.77%) were triple-negative breast cancer (TNBC). In total, 737 (90.7%) patients received postoperative chemotherapy, 460 (56.6%) received postoperative radiotherapy, and 670 (82.5%) received endocrinetherapy. Mean follow-up periods were 56.72 ± 20.29, 58.26 ± 19.86, and 56.10 ± 20.44 months in the whole population, SLNB cohort, and SLNB + ALND cohort.

Compared with patients without ALND, patients with ALND had a significantly increased likelihood of positive SLNs (1.09 ± 0.30 vs. 1.29 ± 0.45, P < 0.001), chemotherapy (85.90% vs. 92.73%, P = 0.002), and radiotherapy (47.44% vs. 60.38%, P < 0.001). No statistically significant differences were observed between the two cohorts for the other factors.

PSM was performed to balance the significant differences between the two cohorts. After the PSM, 234 cases who received ALND and 234 who did not receive ALND were matched. Matching was based on status of ER and PR, HER2, and expression of Ki-67, and the presence or absence of chemotherapy, radiotherapy, and endocrinetherapy. No notable discrepancies were identified in the clinicopathological characteristics between the two cohorts after matching. **Table 1** presents a descriptive characterization of the population.

**Table 1:** Patient clinical and pathological characteristics.

Variable	Before PSM					After PSM				
	Total (n = 812)	SLNB (n = 234)	SLNB+ALND (n = 578)	P	SMD	Total (n = 468)	SLNB (n = 234)	SLNB+ALND (n = 234)	P	SMD
Age, Mean ± SD	51.83 ± 11.16	52.26 ± 12.18	51.66 ± 10.72	0.490	-0.056	51.88 ± 11.72	52.26 ± 12.18	51.51 ± 11.26	0.208	0.124
cT, n (%)				0.066					0.457	
1	410 (50.49)	130 (55.56)	280 (48.44)		-0.142	257 (54.91)	130 (55.56)	127 (54.27)		-0.069
2	402 (49.51)	104 (44.44)	298 (51.56)		0.142	211 (45.09)	104 (44.44)	107 (45.73)		0.069
Lymph vascular invasion, n (%)				0.641					0.809	
Negative	651 (80.17)	190 (81.20)	461 (79.76)		-0.036	377 (80.56)	190 (81.20)	187 (79.91)		0.023
Positive	161 (19.83)	44 (18.80)	117 (20.24)		0.036	91 (19.44)	44 (18.80)	47 (20.09)		-0.023
Perineural invasion, n (%)				0.562					0.524	
Negative	681 (83.87)	199 (85.04)	482 (83.39)		-0.044	398 (85.04)	199 (85.04)	199 (85.04)		-0.058
Positive	131 (16.13)	35 (14.96)	96 (16.61)		0.044	70 (14.96)	35 (14.96)	35 (14.96)		0.058
Histological grade, n (%)				0.293					0.270	
I	48 (5.91)	14 (5.98)	34 (5.88)		-0.004	28 (5.98)	14 (5.98)	14 (5.98)		0.105
II	623 (76.72)	187 (79.91)	436 (75.43)		-0.104	377 (80.56)	187 (79.91)	190 (81.20)		-0.137
III	141 (17.36)	33 (14.10)	108 (18.69)		0.118	63 (13.46)	33 (14.10)	30 (12.82)		0.080
ER, n (%)				0.558					0.779	
Negative	106 (13.05)	28 (11.97)	78 (13.49)		0.045	50 (10.68)	28 (11.97)	22 (9.40)		0.026
Positive	706 (86.95)	206 (88.03)	500 (86.51)		-0.045	418 (89.32)	206 (88.03)	212 (90.60)		-0.026
PR, n (%)				0.579					0.896	
Negative	127 (15.64)	34 (14.53)	93 (16.09)		0.042	63 (13.46)	34 (14.53)	29 (12.39)		0.012
Positive	685 (84.36)	200 (85.47)	485 (83.91)		-0.042	405 (86.54)	200 (85.47)	205 (87.61)		-0.012
HER2, n (%)				0.948					0.951	
Negative	577 (71.06)	168 (71.79)	409 (70.76)		-0.023	341 (72.86)	168 (71.79)	173 (73.93)		0.029
Positive	176 (21.67)	49 (20.94)	127 (21.97)		0.025	87 (18.59)	49 (20.94)	38 (16.24)		-0.021
Uncertain	59 (7.27)	17 (7.26)	42 (7.27)		0.000	40 (8.55)	17 (7.26)	23 (9.83)		-0.018
Ki-67, n (%)				0.954					0.227	
≤14%	383 (47.17)	110 (47.01)	273 (47.23)		0.004	227 (48.5)	110 (47.01)	117 (50.00)		0.112
>14%	429 (52.83)	124 (52.99)	305 (52.77)		-0.004	241 (51.5)	124 (52.99)	117 (50.00)		-0.112
Type, n (%)				0.991					0.560	
Luminal A	310 (38.18)	87 (37.18)	223 (38.58)		0.016	211 (45.09)	96 (41.03)	115 (49.15)		0.148
Luminal B	271 (33.37)	83 (35.47)	188 (32.53)		-0.038	141 (30.13)	73 (31.20)	68 (29.06)		-0.129
Triple-negative	55 (6.77)	15 (6.41)	40 (6.92)		-0.005	23 (4.91)	13 (5.56)	10 (4.27)		-0.020
HER2 positive	176 (21.68)	49 (20.94)	127 (21.97)		0.025	93 (19.87)	52 (22.22)	41 (17.52)		-0.021
Chemotherapy, n (%)				0.002					1.000	
No	75 (9.24)	33 (14.10)	42 (7.27)		-0.263	48 (10.26)	33 (14.10)	15 (6.41)		0.000
Yes	737 (90.76)	201 (85.90)	536 (92.73)		0.263	420 (89.74)	201 (85.90)	219 (93.59)		0.000
Radiotherapy, n (%)				<.001					0.642	
No	352 (43.35)	123 (52.56)	229 (39.62)		-0.265	217 (46.37)	123 (52.56)	94 (40.17)		0.043
Yes	460 (56.65)	111 (47.44)	349 (60.38)		0.265	251 (53.63)	111 (47.44)	140 (59.83)		-0.043
Endocrinotherapy, n (%)				0.987					0.904	
No	142 (17.49)	41 (17.52)	101 (17.47)		-0.001	72 (15.38)	41 (17.52)	31 (13.25)		0.011
Yes	670 (82.51)	193 (82.48)	477 (82.53)		0.001	396 (84.62)	193 (82.48)	203 (86.75)		-0.011

SLNB, sentinel lymph node biopsy; ALND, axillary lymph node dissection; PSM, propensity-score matching; SD, standard deviation; ER, estrogen receptor; PR, progesterone receptor; HER2, human epidermal growth factor receptor 2.

### 3.2 Survival analyses of the whole population

**Figure 2:**
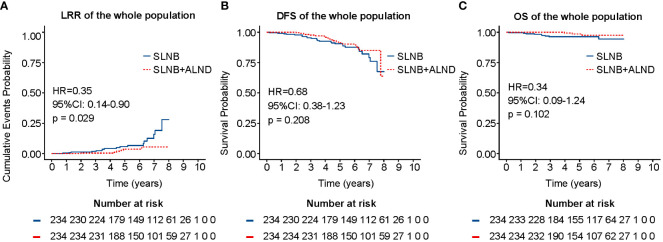
LRR **(A)**, DFS **(B)**, and OS **(C)** of the whole population after PSM. LRR, locoregional recurrence rate; DFS, disease-free survival; OS, overall survival; PSM, propensity score matching.

As shown in **Figures 2B, C**, the SLNB + ALND cohort exhibited no notable disparity in DFS (HR = 0.68, 95% CI: 0.38 - 1.23, P = 0.208) or OS (HR = 0.34, 95% CI: 0.09 - 1.24, P = 0.102) in comparison to SLNB cohort. However, the LRR was found to be distinctly less prevalent in the SLNB + ALND cohort in comparison to the SLNB cohort (2.76% vs. 7.83%, P = 0.029) (**Figure 2A**). In univariate cox analysis, age, axillary surgery, ER, PR, Ki-67, and endocrinotherapy were associated with LRR (**Supplementary Figure 1**). Subsequent multivariate cox proportional hazards analysis revealed that younger (≤ 40 years) (HR = 3.72, 95% CI: 1.36 - 10.14, P = 0.01), progesterone receptor-negative (HR = 3.92, 95% CI: 1.65 - 9.34, P = 0.002), and SLNB alone (HR = 2.56, 95% CI: 1.01- 6.67, P = 0.046) were related to an increased risk of LRR. Univariate cox analysis for DFS and OS can be seen in **Supplementary Figures 2**, **3**. In general, the multivariate analysis demonstrated that cT2 (HR = 3.61, 95% CI: 1.36 - 9.61, P = 0.01) and perineural invasion (HR = 10.21, 95% CI: 3.95 - 26.37, P < 0.001) were predictive risk factors, while endocrinotherapy (HR = 0.08, 95% CI: 0.02 - 0.27, P < 0.001) was a beneficial prognostic indicator for OS among patients with those hormone receptor positive, similar phenomena could be observed in DFS.

### 3.3 Survival analyses in four molecular subtypes

**Figure 3:**
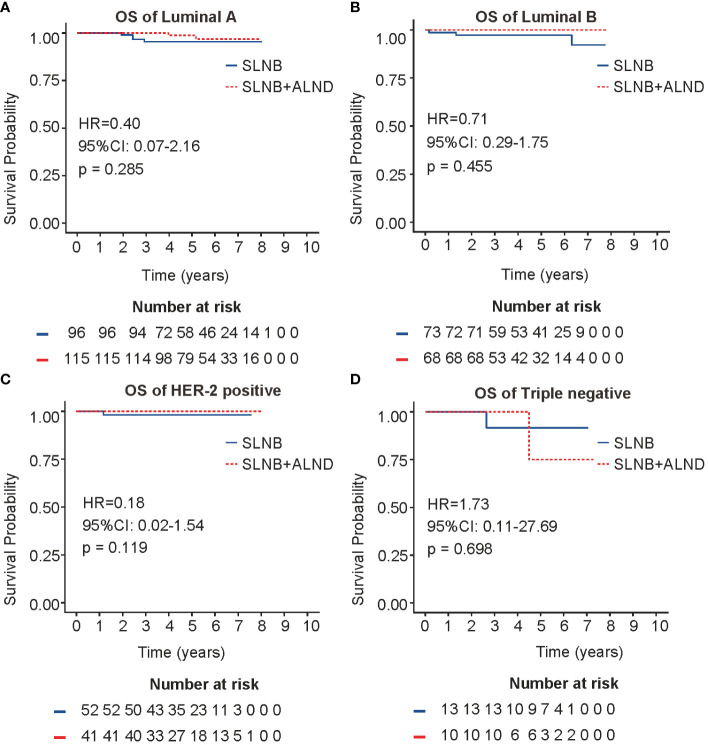
OS in four molecular subtypes. **(A)** OS of Luminal A breast cancer. **(B)** OS of Luminal B breast cancer. **(C)** OS of HER2 positive breast cancer. **(D)**. OS of Triple negative breast cancer. OS, overall survival.

To explore the survival prognosis of ALND in different molecular subtypes (luminal A, luminal B, HER2 positive and TNBC), a Kaplan-Meier analysis was conducted. As depicted in **Figures 3A, B**, no statistical differences in overall survival were observed for patients in the SLNB + ALND cohort when compared to those in the SLNB cohort with luminal A (HR = 0.40, 95% CI: 0.07 - 2.16, P = 0.285) or luminal B (HR = 0.71, 95% CI: 0.29 - 1.75, P = 0.455) breast cancer. As presented in **Figure 3C**, a comparable situation was detectable in the HER2 positive. In addition, patients in the SLNB + ALND cohort had similar OS (HR = 1.73, 95% CI: 0.11 - 27.69, P = 0.698) (**Figure 3D**) and DFS (HR = 1.30, 95% CI: 0.25 - 6.67, P = 0.753) (**Supplementary Figure 4**) to those in the SLNB cohort with TNBC. Moreover, in the HER2 positive, patients in the ALND cohort had a DFS advantage (HR = 0.27, 95% CI: 0.06 - 1.30, P = 0.103) (**Supplementary Figure 4**), while not reaching statistical significance.

### 3.4 Stratification analyses by age groups

**Figure 4:**
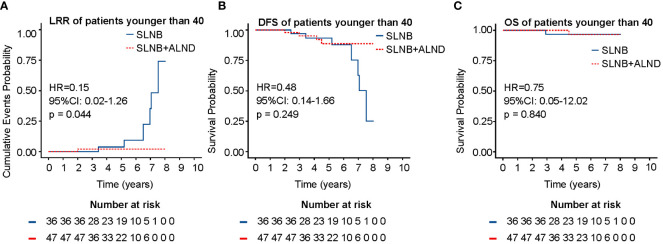
LRR **(A)**, DFS **(B)**, and OS **(C)** of patients younger than 40 years. LRR, locoregional recurrence rate; DFS, disease-free survival; OS, overall survival.

The whole cohort was divided into three groups by age: ≤ 40 years, 41 - 55 years, and ≥ 56 years. Further analysis showed that in patients ≤ 40 years, ALND did not improve DFS (HR = 0.48, 95% CI: 0.14 - 1.66, P = 0.249) or OS (HR = 0.75, 95% CI: 0.05 - 12.02, P = 0.840), but it decreased LRR (11.1% vs. 2.12%, P = 0.044) in this group (**Figure 4**). However, no difference in survival and LRR was found between the SLNB + ALND and SLNB groups in patients aged 41-55 years and ≥ 56 years (**Supplementary Figure 5**).

## 4 Discussion

A tendency of de-escalation has been noticed in the axillary surgery of breast cancer. Whether ALND can be omitted in mastectomy remains controversial. The percentage of ALNDs has been gradually reducing in recent years (15). Additionally, patients who had breast-conserving were more often to avoid ALND than those who had a mastectomy (22). In our research, stratified according to the age and molecular subtypes, we examined the impacts of different treatment strategies of axillary on the prognosis of 1-2 metastatic SLNs patients who had a mastectomy. We found that patients in the SLNB cohort had comparable OS and DFS with those in the SLNB + ALND cohort in four molecular subtypes. In the ≤ 40 years group, the ALND cohort had a lower LRR. No differences in survival and LRR between the SLNB + ALND and SLNB were found in other age groups.

In the whole population, the proportion of patients receiving chemotherapy and radiotherapy was significantly higher in ALND cohort than SLNB cohort. Using adjuvant chemotherapy and radiotherapy may affect outcomes when analyzing patients with or without ALND (23, 24). Matching factors were ER status, PR status, HER2 status, Ki-67, and the presence or absence of chemotherapy, radiotherapy, and endocrinetherapy. It should be noted that assessing the precise effect of adjuvant treatments on axillary surgery remains challenging, despite the outlined efforts. Due to the inherent limitations of PSM, despite matching based on these indicators, it is undeniable that there may still be unmatched variables that could lead to biased results.

For the whole population, no significant differences in LRR, DFS and OS were observed between the ALND and SLNB cohorts. However, subgroup analysis by age revealed that in individuals younger than 40 years, the ALND cohort exhibited lower LRR rates compared to the SLNB cohort, with no significant differences in DFS and OS. This suggests that locoregional recurrence may be more likely to occur in certain subgroups, such as younger individuals, which is corroborated by the findings of our multivariate analysis. In this analysis, younger individuals (≤ 40 years) were identified as an independent contributing factor to LRR.

We successfully matched 234 patients in each of the two cohorts by PSM. The effects of treatment factors, including chemotherapy, radiotherapy, and endocrinetherapy on LRR, DFS, and OS were negated by PSM. Following this adjustment, no distinction was observed in the percentage of patients treated with radiotherapy among the two cohorts. The results indicate that for the whole population, patients without ALND did not experience worse LRR, DFS, or OS. The multivariate analysis showed that being younger (≤ 40 years), progesterone receptor-negative, and undergoing SLNB alone were independent risk factors for LRR. Considering this result, we recommend that for patients without high-risk factors, axillary interventions such as ALND or radiotherapy may be avoided.

The results of previous studies have reported similar DFS, OS, and LRR among patients with SLNB alone (25). The results of our investigation revealed no meaningful statistical differences in either OS or DFS between the two cohorts. These findings align with those reported by the study carried out by Susan B. Kesmodel et al. (13, 16, 26). The subsequent analysis demonstrated that patients aged ≤ 40 years in the SLNB + ALND cohort experienced a considerably lower rate of LRR than those in the SLNB cohort. However, no statistical difference was found in the ten-year regional recurrence between ALND and SLNB cohorts (8).

Studies have reported associations between HER2 poitive and TNBC breast cancer subtypes with worse survival and higher rates of LRR (27, 28). However, this feature was not found in our study. Therefore, further investigation is necessary to validate these conclusions.

The use of molecular subtypes as the basis for selecting treatments and predicting prognosis is becoming a more prevalent practice in oncology. It is generally believed that Luminal type breast cancer is sensitive to endocrinetherapy and has a better prognosis than other types (29). HER2 positive patients are sensitive to target therapy, while patients with triple-negative breast cancer lack individualized treatment plans. HER2 positive and TNBC patients have a more unfavorable prognosis. Patients with luminal A and TNBC have a less significant likelihood of axillary lymph node involvement, whereas luminal B and HER2 positive demonstrate a higher incidence of axillary lymph node positive. Although our analysis did not show a survival benefit of ALND for patients with HER2 positive or luminal B, considering their unique biological behavior, we still recommend that clinicians adopt a more aggressive approach to axillary management for these patients.

The biology of breast cancer, rather than the scope of surgical intervention, has been acknowledged as a significant determinant of systemic and locoregional recurrence risk (8). We found that cT1, receipt of endocrinotherapy, and negative perineural invasion were correlated with better OS and DFS. Studies have shown that tumors exhibiting low expression of PR demonstrated more aggressive behaviors compared to those with high expression (30). A study by Arisio et al. demonstrated that hormone therapy significantly impacted OS and DFS (31). Additionally, research by Giuliano et al. has indicated that hormone receptor status, age at disease diagnosis, and the management of adjuvant therapy were related to OS, which is consistent with our findings (8, 32). When perineural invasion is detected in mastectomy patients, it is recommended to administer aggressive post-surgical treatment and conduct an intensive follow-up program (26).

After stratifying patients by age, it was observed that for those aged 41 years and above, ALND did not provide significant survival benefits. This is consistent with the trend of de-escalated treatment in elderly patients (33–35). Despite our population being considered younger than the criteria in previous studies, which classified patients aged 70 years and above as elderly (36). The study by Gu et al. suggested that the exclusion of axillary surgery was not correlated with inferior OS but was correlated with an increased incidence of LRR (37). Interestingly, a similar phenomenon was observed in patients aged ≤ 40 years in our study. However, age did not exhibit a significant association with LRR after adjusting for other factors in ACOSOG Z0011 (20). It has been documented that LRR events occur within the initial five-year period following the commencement of treatment, a critical factor in the management of breast cancer (38, 39).

In our research, there were 10 cases (1.2%) of invasive lobular carcinoma (ILC) and 7 cases (0.8%) of invasive ductal carcinoma (IDC) concomitant with ILC. Given the limited sample size and the fact that, clinically, systemic treatments rarely distinguish between these two histological types, we have not provided a detailed analysis of this category. However, it is undeniable that ILC and IDC have some differences in their biological behavior. For example, ILC tends to have larger tumor sizes, a higher incidence of multifocality, and is more common in Luminal A, which are characteristics associated with a favorable prognosis. There is inconsistent evidence in the literature regarding the outcomes of ILC compared to those of IDC (40). In our future research, we plan to expand the sample size and discuss the differences in survival prognosis and other aspects between this type of breast cancer and IDC.

It should be noted that the study is not without its inherent limitations. Firstly, given the retrospective data collection, there is a potential for selection bias that cannot be overlooked. Secondly, as complete data on molecular targeted therapy were not available, it was impossible to analyze how anti-HER2 therapy impact clinical outcomes in these patients. Thirdly, a relatively short follow-up duration may introduce bias in the survival analysis of axillary surgical management.

## 5 Conclusions

Our study found that the younger (≤ 40 years), PR-negative, and SLNB alone were independent risk factors for LRR. Perineural invasion was an independent risk factor, while endocrinotherapy was a beneficial prognostic indicator for DFS and OS among patients with positive hormone receptor. ALND does not impact DFS and OS in patients with 1-2 metastatic SLNs who have completed a mastectomy and may be considered to be omitted in patients without risk factors.

## Data availability statement

The original contributions presented in the study are included in the article/**Supplementary Material**. Further inquiries can be directed to the corresponding author.

## Ethics statement

The studies involving humans were approved by The Ethics Committee of The Fourth Hospital of Hebei Medical University (2022KY054). The studies were conducted in accordance with the local legislation and institutional requirements. Written informed consent for participation was not required from the participants or the participants’ legal guardians/next of kin in accordance with the national legislation and institutional requirements.

## Author contributions

XZ: Conceptualization, Formal analysis, Investigation, Methodology, Resources, Visualization, Writing – original draft, Writing – review & editing. LY: Formal analysis, Investigation, Methodology, Visualization, Writing – original draft, Writing – review & editing. CC: Investigation, Writing – review & editing, Methodology. ZS: Investigation, Writing – review & editing, Conceptualization, Funding acquisition, Project administration, Supervision, Validation.
