# Supplementary material for: The prognostic analysis of further axillary dissection in breast cancer with 1-2 positive sentinel lymph nodes undergoing mastectomy
Source: Front Oncol. 2024 Aug 5;14:1406981. doi: 10.3389/fonc.2024.1406981 (PMC11330764; doi:10.3389/fonc.2024.1406981)
Supplement: Supplementary file 1 [file DataSheet_1.zip › supplementary_material/Supplementary_Material.docx]

Supplementary Material

S1 Fig. Univariate cox regression model forest graph of locoregional recurrence rate (LRR).

S2 Fig. Univariate cox regression model forest graph of disease-free survival (DFS).

S3 Fig. Univariate cox regression model forest graph of overall survival (OS).

S4 Fig. DFS in four molecular subtypes. **(A)**. DFS of Luminal A breast cancer. **(B)**. DFS of Luminal B breast cancer. **(C)**. DFS of HER2 positive breast cancer. **(D)**. DFS of Triple negative breast cancer. DFS, disease-free survival.

S5 Fig. LRR **(A, D)**, DFS **(B, E)**, and OS **(C, F)** of 41-55 years **(A-C)** and older than 56 years **(D-F)** breast cancer patients. LRR, locoregional recurrence rate; DFS, disease-free survival; OS, overall survival.
