# Supplementary figures and images for: The prognostic analysis of further axillary dissection in breast cancer with 1-2 positive sentinel lymph nodes undergoing mastectomy
Source: Front Oncol. 2024 Aug 5;14:1406981. doi: 10.3389/fonc.2024.1406981 (PMC11330764; doi:10.3389/fonc.2024.1406981)

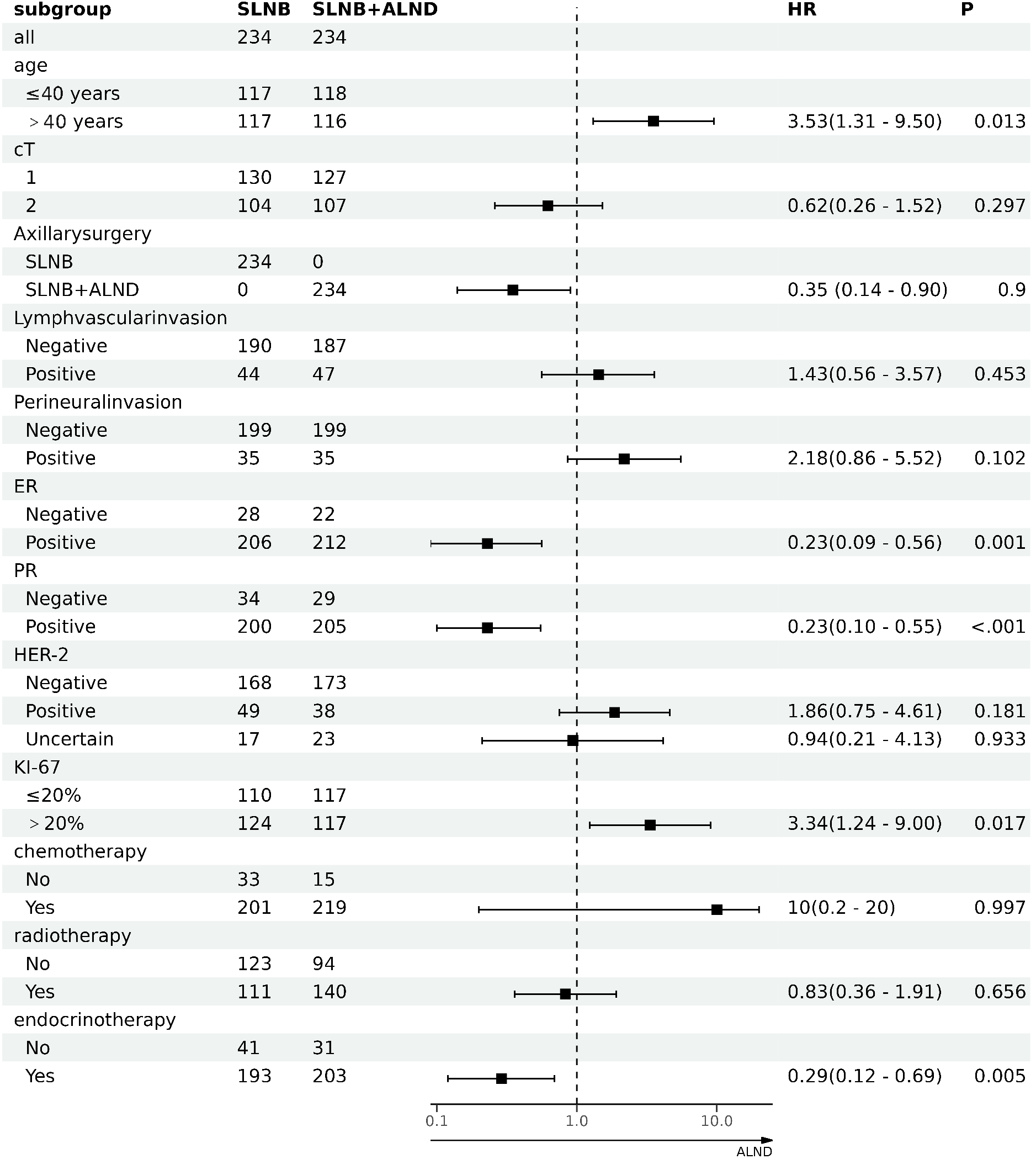

Supplement: Supplementary file 1 [file DataSheet_1.zip › supplementary_material/S1_Fig.tif]

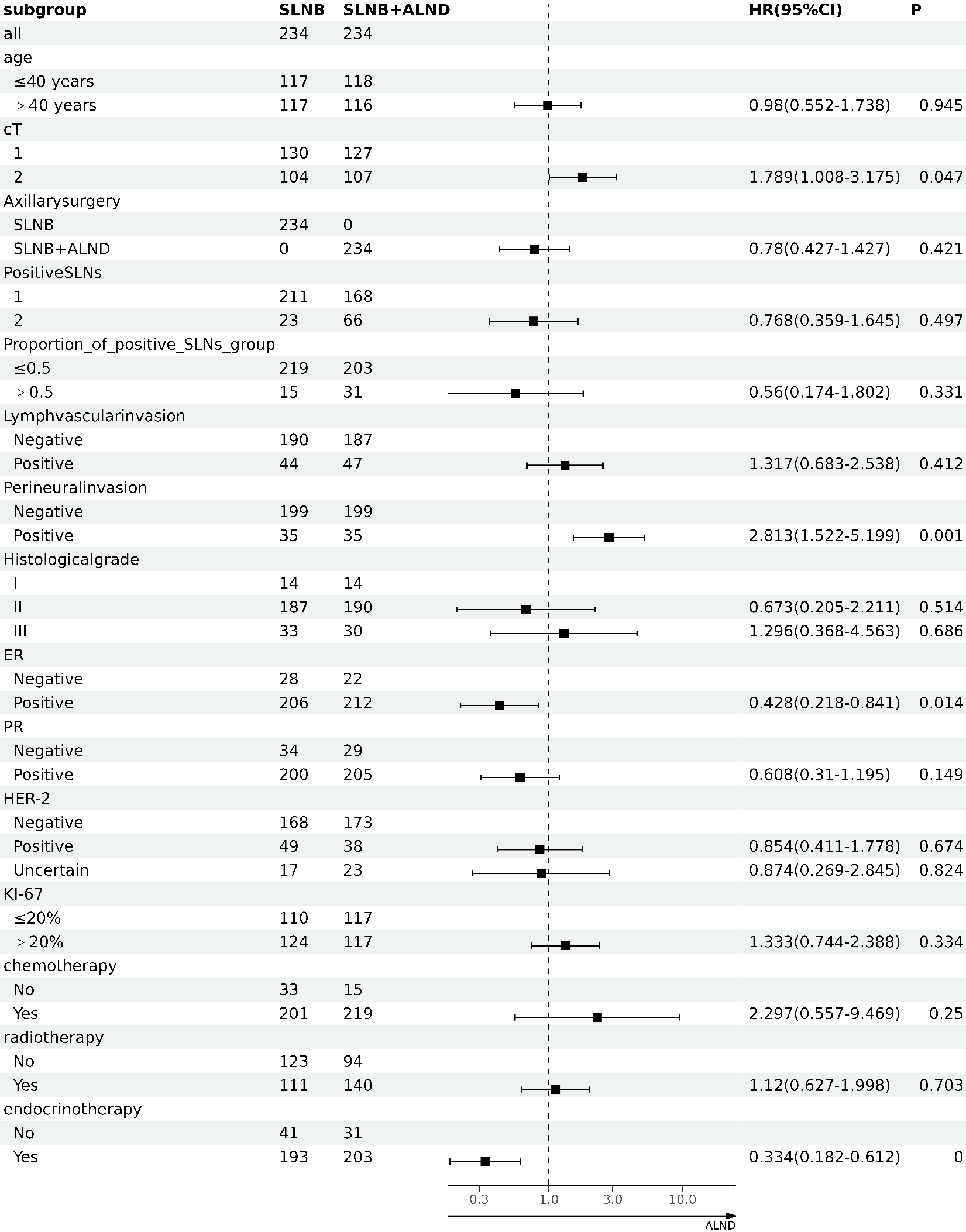

Supplement: Supplementary file 1 [file DataSheet_1.zip › supplementary_material/S2_Fig.tif]

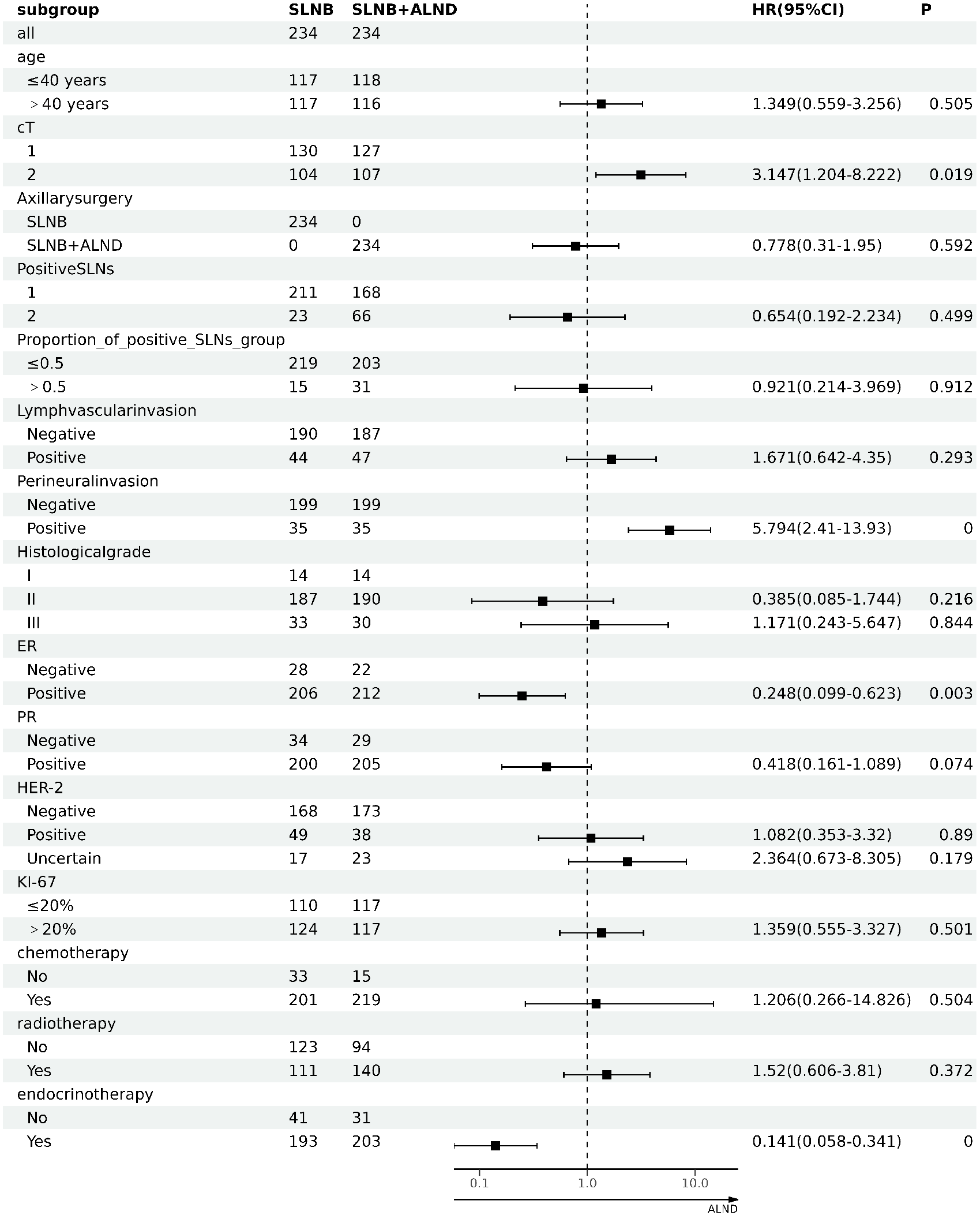

Supplement: Supplementary file 1 [file DataSheet_1.zip › supplementary_material/S3_Fig.tif]

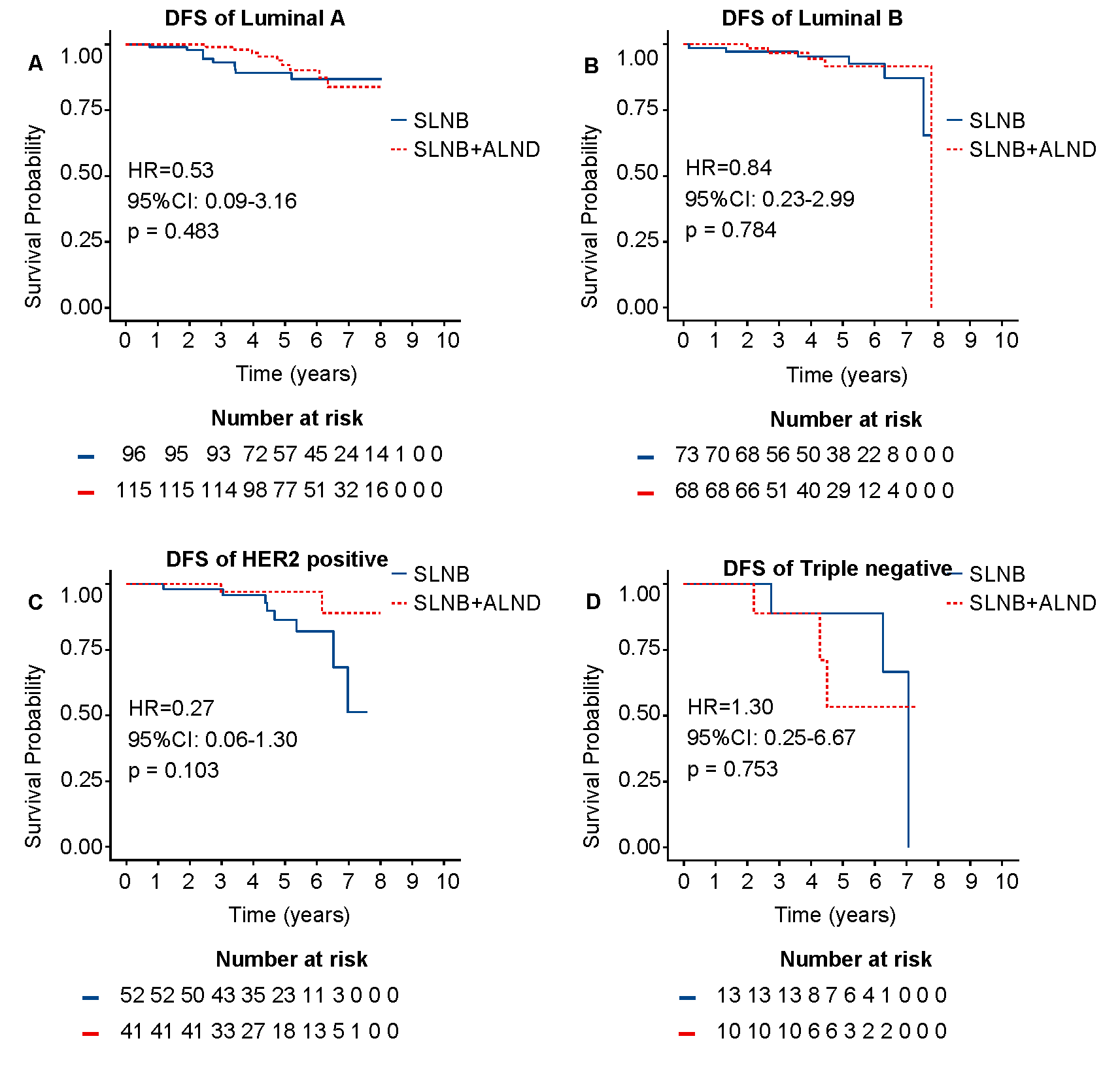

Supplement: Supplementary file 1 [file DataSheet_1.zip › supplementary_material/S4_ Fig.tif]

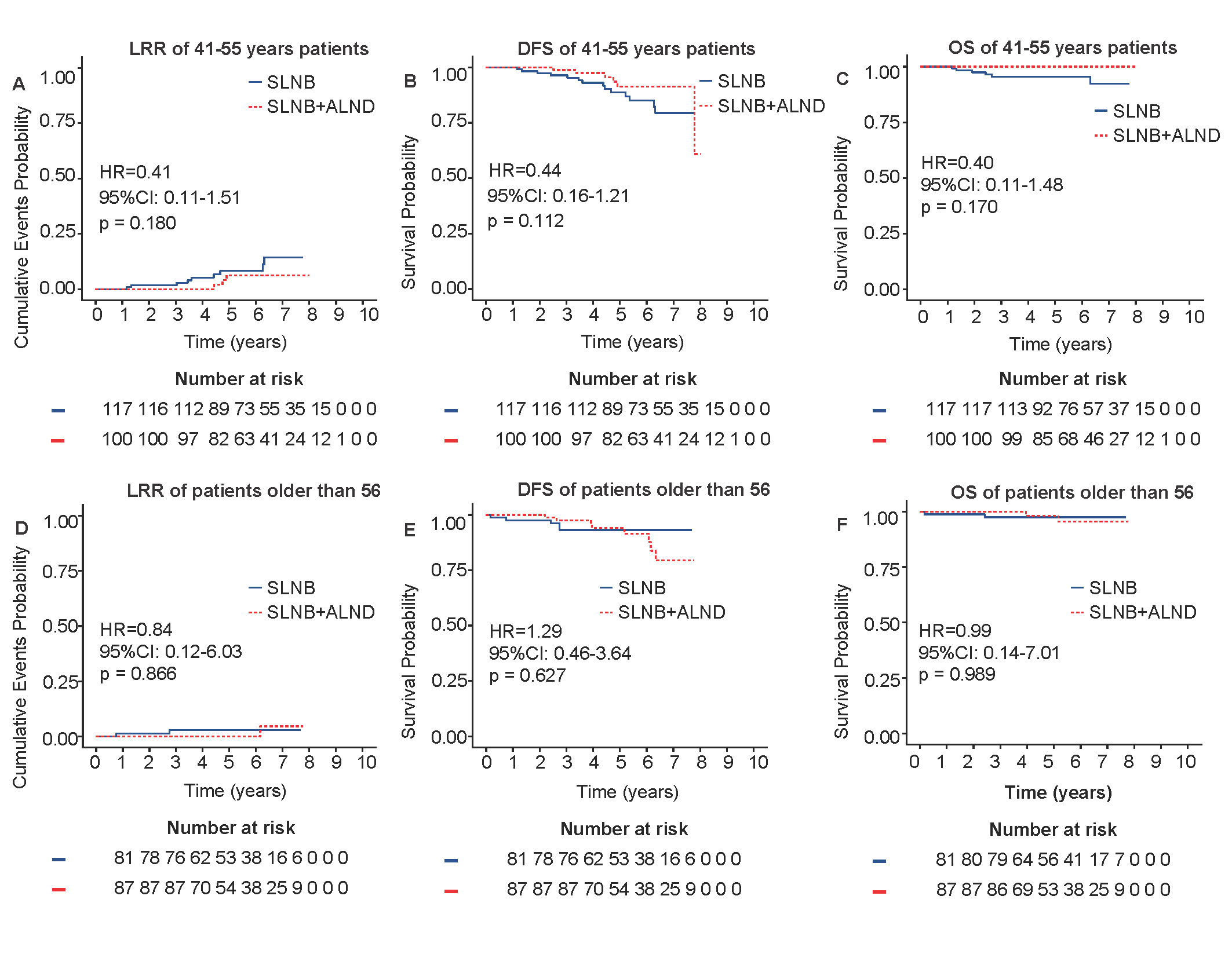

Supplement: Supplementary file 1 [file DataSheet_1.zip › supplementary_material/S5_ Fig.tif]
